# Boat encounter with the 2019 Java bioluminescent milky sea: Views from on-deck confirm satellite detection

**DOI:** 10.1073/pnas.2207612119

**Published:** 2022-07-11

**Authors:** Steven D. Miller

**Affiliations:** ^a^Department of Atmospheric Science and Cooperative Institute for Research in the Atmosphere, Colorado State University, Fort Collins, CO 80523-1375

**Keywords:** bioluminescence, microbial ecology, oceanography, satellite, remote sensing

## Abstract

“Milky seas” are massive swaths of uniformly and steadily glowing ocean seen at night. The phenomenon is thought to be caused by luminous bacteria, but details of milky sea composition, structure, cause, and implications in nature remain largely uncertain. Between late July and early September 2019, specialized low-light satellite sensors detected a possible bioluminescent milky sea south of Java, Indonesia, spanning >100,000 km^2^. Upon learning of these findings, crew members of the yacht *Ganesha* reached out to confirm and share details of their personal encounter with this same event. Here, we document *Ganesha*’s experience as recalled by the crew, compare their course to satellite data, and assess their photography of this milky sea.

Milky seas (1) are a rare (approximately zero to two times per year globally) form of marine bioluminescence which impart to the nighttime ocean surface the surreal appearance of a daylit snowfield under dark, moonless skies. Sailors’ reports over the centuries suggest milky seas occur preferably in the northwestern Indian Ocean and Maritime Continent region. Unlike the transient flashes of bioluminescence made by phytoplankton in disturbed waters, milky seas produce a steady glow, even in calm waters. They are thought to be caused by luminous bacteria, communicating with each other and triggering a glowing response upon reaching critical populations via a process called quorum sensing (2).

Milky seas have eluded scientific inquiry due to their remote, transient, and infrequent nature. In 1985, a US naval research vessel happened upon one in the Arabian Sea, near Socotra (3). A British merchant vessel crossed another offshore of Somalia in 1995, confirmed years later by a satellite-based low-light sensor, the Operational Linescan System (OLS) (4). While these cases offered important insights on the composition and structure of milky seas, answers to detailed questions await rigorous in situ sampling, and a practical way of locating events in progress.

A new-generation instrument on National Oceanic and Atmospheric Administration (NOAA) environmental satellites is lighting a path forward. Members of NOAA’s Joint Polar Satellite System (JPSS) (5) carry the Day/Night Band (DNB) (6), an imager capable of detecting light a billion times fainter than sunlight. A recent 10-y survey of DNB imagery yielded 12 milky sea candidates, including a large event (>100,000 km^2^) south of Java in July–September 2019 (7). However, those detections lacked surface corroboration.

That confirmation is now in hand, thanks to a chance encounter with the 2019 Java milky sea by the crew of *Ganesha*, a 16-m private yacht, who reported crossing through a swath of brightly glowing waters on the night of 2 August 2019. Unfamiliar with the milky sea phenomenon, they were flummoxed until one of the crew (Naomi McKinnon) learned, via media coverage, of the satellite detections. The *Ganesha* account provides an opportunity to relate satellite data to surface observations, including raw photography of the waters aglow.

## Results

Captained by Johan Lemmens with six other crew aboard, *Ganesha* was embarked on a circumnavigation of the globe when it crossed the 2019 Java milky sea midway in its leg between Lombok, Indonesia, and the Cocos (Keeling) Islands in the east Indian Ocean. Their experience began on the night of 2 August at ∼2100 local time (∼1400 Greenwich Mean Time [GMT]) and lasted until dawn the following morning (a total traverse time of ∼8 h). An entry from *Ganesha’s* ship log at 2200 local time, reads,

When waking up at 2200 the sea was white. There is no moon, the sea is apparently full of ? plankton ? but the bow wave is black! It gives the impression of sailing on snow!

Interviews with the crew yielded additional details. *Ganesha* entered these glowing waters suddenly, and, thereafter, the entire ocean was significantly brighter than the night sky—maintaining a mostly homogenous and steady glow to the horizon. A bucket sample of these waters, whose collection did not disrupt the illumination at that location, contained several pinpoints of steady glow that darkened upon stirring—a behavior opposite to that of “normal” bioluminescence. Likewise, the crew noted a darkened bow wave, but the ship wake had no perceivable change in brightness from the surrounding glowing waters.

**Fig. 1.**
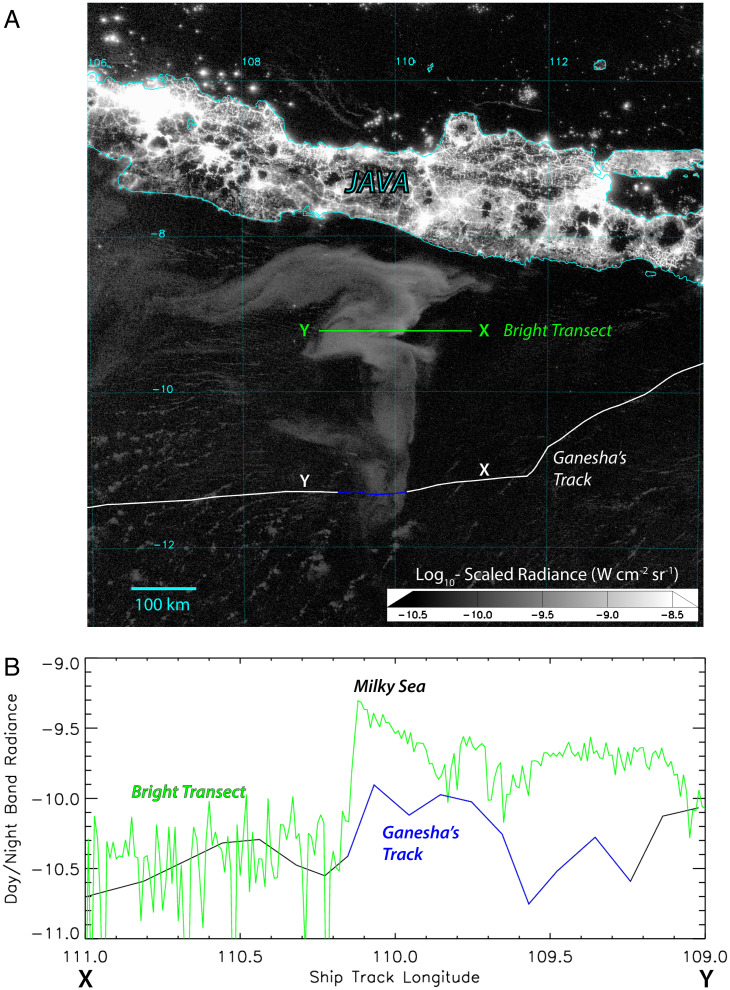
(*A*) DNB imagery of the 2019 Java milky sea on 2 August 2019 at 1752 Coordinated Universal Time, showing a ∼100,000 km^2^ swath of glowing ocean. (*B*) Transects of *Ganesha*’s track and a cut through the brightest portion of the milky sea between longitudinal positions X→Y are shown, along with corresponding DNB radiance (watts per square centimeter per steradian, log_10_-scaled) cross-sections.

Satellite imagery collected on 2 August 2019 at 1752 GMT (Fig. 1*A*), roughly midway through *Ganesha’*s encounter, shows the milky sea as a diffuse “anvil-shaped” feature between 8°S to 12°S and 107°E to 111°E. A multinight animation of *Ganesha*’s course is provided as Movie S1. Overlaid as a white line is the vessel’s track, per GPS data, with the locations at reported times of their encounter shown as a blue segment, illustrating how the vessel transected the southern extremity of the feature. Weather conditions leading up to the encounter were fair, with a moderate (11 kt to 16 kt) easterly breeze, slight seas, and good visibility. By 0500 local time the next morning, conditions calmed to light (4 kt to 6 kt) breeze, smooth seas, and visibility listed playfully in the logbook as “glowing ☺.”

Extraction of DNB radiance data along *Ganesha*’s path (Fig. 1*B*) shows elevated values upon entry into the milky sea (blue portion of line, corresponding to Fig. 1*A*). Notably, *Ganesha* did not cross the most intensely glowing waters; an area ∼200 km to the north of its transect produced radiances 4 to 5 times greater. For comparison, a hypothetical crossing through that brighter area is shown as a green line in Fig. 1 *A* and *B*.

**Fig. 2.**
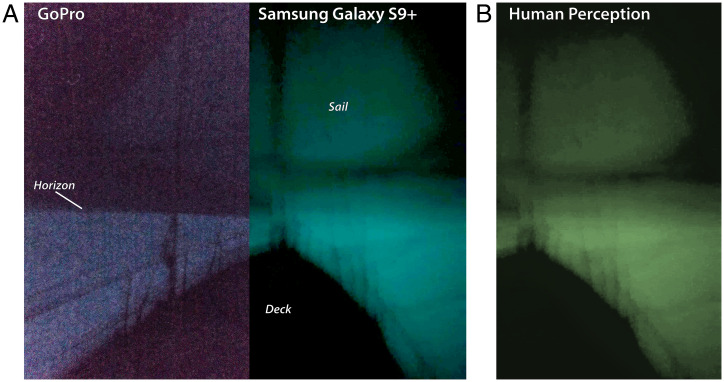
Digital photography of the 2019 Java milky sea, captured by *Ganesha*’s crew, showing a view of (*A*) the ship’s prow and (*B*) a color-adjusted version of the Samsung photo approximating the visual perception of the glow. The ocean’s state was calm.

The crew attempted photography of the milky sea via a Go-Pro camera and a higher-quality Samsung Galaxy S9+ phone camera. Fig. 2 is a port/starboard splice of the two photos, facing toward the ship’s prow (pointing west). The saturation in these digital images was increased to an intensity consistent with the crew’s recalled perception. Both cameras captured the essence of the ocean’s widespread glow in contrast to dark sky along the horizon. *Ganesha*’s deck and railings appear dark, while the sail reflects the light upwelling from the ocean. These photos give visual testimony to the written accounts of mariners across the centuries.

## Discussion

Although *Ganesha*’s logbook likened the glow to a white snowfield, interviews with the captain and crew indicated that coloration was indeed present, as implied by the photography. One insightful description follows:

Both the color and intensity of the glow was akin to glow-in-the-dark stars/stickers, or some watches that have glowing parts on the hands … a very soft glow that was gentle on the eyes.

The perception of color varied slightly between the witnesses, attributable, perhaps, to the varying responses of the eye on the fringes between scotopic and photopic vision under low-light conditions. The hue values in Fig. 2*A* were unaltered—displaying the original color as sensed by the cameras. In Fig. 2*B*, the Samsung photo was modified to approximate the human perception as suggested above.

It was the impression of Captain Lemmens that the glow originated from some depth (∼10 m) below the surface. Combined with the lack of a darkened ship wake, these observations appear to contradict the surface slick hypothesis (3) for milky sea structure, which postulates that the luminous bacteria colonize only a thin film that is disrupted by a vessel’s hull. The behavior of *Ganesha*’s bucket sample and dark bow waves, where enhanced mixing suppressed the apparent bioluminescence, favor, instead, a volumetric (7) and/or submerged (8) bacterial emission source. Thus it remains unclear whether the presence/absence of darkened ship wakes relates to fundamental differences in milky sea morphology, or whether variances observed within a common morphology are explainable, for example, by the size and water-stirring properties of different vessels. Such questions could be answered via detailed vertical profiles of milky sea water properties, organic content, and luminosity.

A significant aspect of *Ganesha*’s encounter is the ordering of events. In the 1995 Somali milky sea case (4), the SS *Lima* report prompted satellite confirmation via OLS imagery, whose poor quality limited its utility to retrospective analyses at best. In the 2019 Java case (7), the higher-quality DNB satellite imagery prompted the surface confirmation. The *Ganesha* account removes any lingering doubt as to the DNB’s capability to detect milky seas.

Many questions related to milky sea structure, composition, and significance await in situ sampling to answer fully. Now, with confirmation of the DNB’s ability to identify milky seas independently from space, we are in a far better position to study them. Understanding milky sea formation in the context of ocean/atmosphere/biosphere coupling can help us anticipate the location and timing of future events. With a newfound confidence in our spaceborne lookouts, a directed expedition to a milky sea enters the realm of possibility.

## Materials and Methods

Ship track data for the *Ganesha* were obtained from PredictWind (https://forecast.predictwind.com/tracking/display/Ganesha/) (9). DNB data are available from NOAA’s Comprehensive Large Array-Data Stewardship System (CLASS; https://www.avl.class.noaa.gov/) (10). Accounts and photography obtained during interviews with Captain Johan Lemmens, and crew members Naomi McKinnon, Coraline Bourdon (GoPro photo), and Leon Schommer (SamSung S9+ photo) are conveyed with their full consent.

## Supplementary Material

Supplementary File

## Data Availability

All study data are included in the article and/or supporting information. Ship track data used in this study are freely available from the PredictWind website (9), and satellite data used in this study are freely available from the NOAA/CLASS website (10).
