# Supplementary figures and images for: Boat encounter with the 2019 Java bioluminescent milky sea: Views from on-deck confirm satellite detection
Source: Proc Natl Acad Sci U S A. 2022 Jul 11;119(29):e2207612119. doi: 10.1073/pnas.2207612119 (PMC9303900; doi:10.1073/pnas.2207612119)

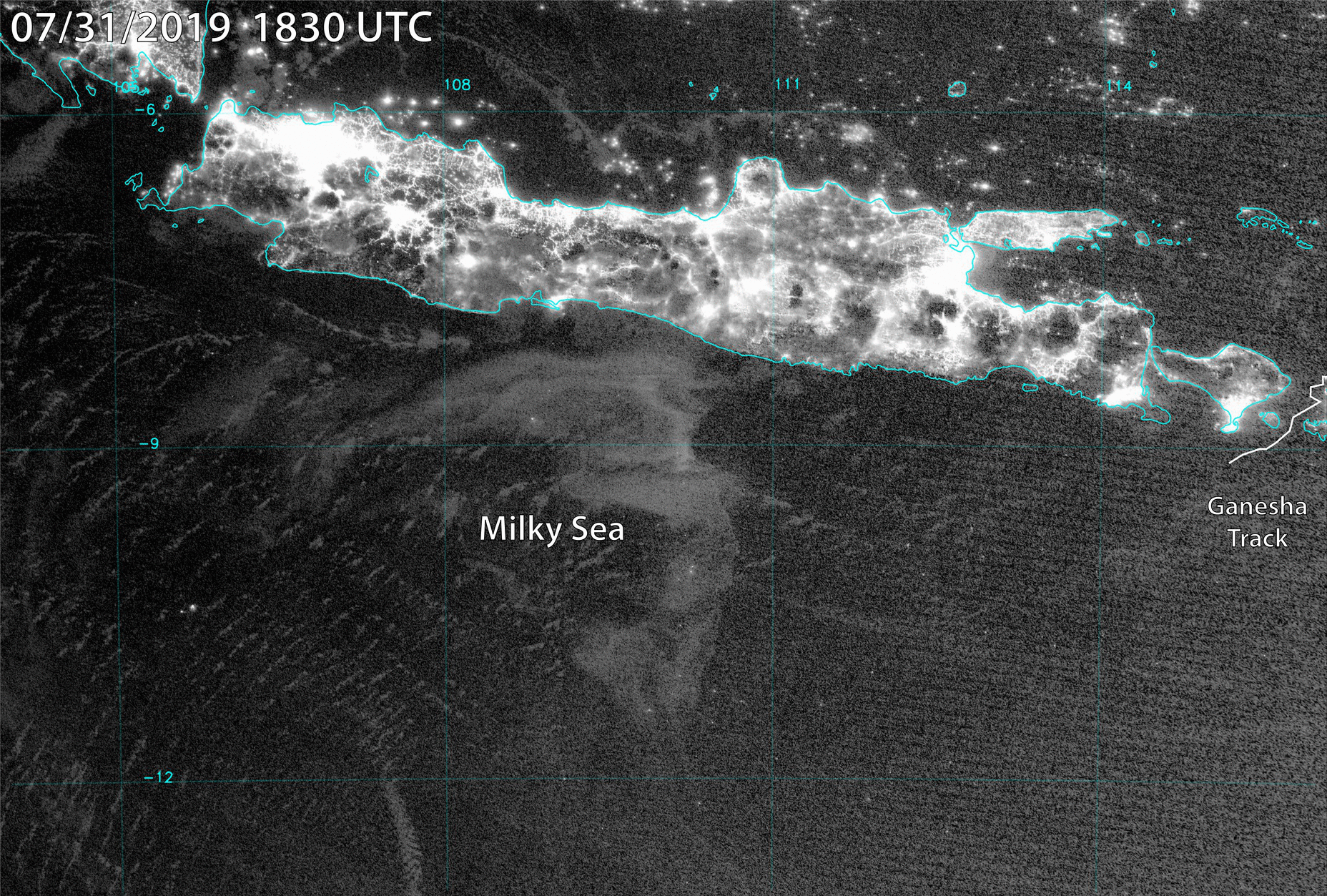

Supplement: Supplementary File [file pnas.2207612119.sm01.gif]
